# Lessons from Brazil: on the difficulties of building a universal health care system

**DOI:** 10.7189/jogh.07.010303

**Published:** 2017-06

**Authors:** Valbona Muzaka

**Affiliations:** King’s College London, London, UK

A number of developing countries that are often referred to as emerging economies have turned their attention to addressing their public health issues in more comprehensive and systematic ways. One of the most notable recent additions to the ranks of these countries is India, where consultations about building a universal health care system have been going on since 2015. While the trajectory of this particular initiative and similar ones elsewhere is yet to be determined, the aim of this piece is to draw some lessons from an emerging economy that, for contingent historical and political reasons, started building a universal public health care system earlier: Brazil. The key argument offered from the Brazilian experience is that building a robust public health care system based on the principles of universality and equity is a challenge of a political economy nature and one that ought to be met at multiple levels simultaneously.

The Indian National Health Policy draft published in 2015 recommended the creation of a universal health care system in India based on equity and universality [1], a progressive move that should be unanimously applauded especially in light of the relatively poor state of health in the country. The challenges of building a public health care system have been enormous wherever this project has been attempted, as any historical overview of the more successful public health care systems would confirm. These challenges are obviously time– and place–specific, as those drafting the Indian National Health Policy seem to be well aware of. One interesting element of this initiative is the relatively delayed response of the Indian state to fulfilling the right to health that, although not explicitly listed as a fundamental right in the Indian Constitution, has been consistently interpreted by the judiciary as central to the right to life guaranteed under Article 21. Another interesting element of this public health initiative relates to its appeal to universality and equality at a time these principles have come under increased pressures – not only of a financial nature – in many existing and well–established public health care systems (eg, in Europe). Both these characteristics – the relatively late emergence of efforts to build a public health care system and doing so upon principles of universality and equity currently under threat everywhere – will no doubt generate daunting challenges to Indian policymakers. It would be futile, of course, to engage in speculation regarding the fate of this policy initiative at this point in time. What would be more productive – and this is the route this article offers – is to attempt to draw lessons from other countries that, like India, also started building their public health care systems relatively late and, like India, face enormous challenges relating to disease burden, inequality in access and quality of health care and a large socially– and economically–excluded part of the population coexisting with a relatively strong and growing private health care industry. One such case is Brazil.

From the early 1920 until the new Constitution of 1988, the Brazilian state had presided over a health care system characterized by a deeply discriminatory principle that restricted medical coverage, and other social rights, to those in the formal job market, excluding agricultural workers, the unemployed and the informal sector workers, in short, the majority of the population [2]. Even during 1950–1980, when Brazil’s economy was growing at an average of nearly 7% per annum, compared to the 5% global annual average and nearly double that of India, the focus of Brazil’s “conservative–informal” welfare regime remained on those groups with the strongest potential to organize politically – ie, the formally employed – while the excluded majority relied on a mixture of familial, philanthropic and meager public care if and when these existed. Healthcare services to the formally employed were based on a dual, private–public financing system and were provided mostly through the private health care sector that started to expand considerably after the military regime came in power in the mid–1960s.

The new Brazilian Constitution of 1988 achieved more than the political inclusion of what had been an excluded majority for the first time in Brazil’s history. The crowning victory of various social movements that had insisted on the simultaneous and universal recognition of political and *socia*l rights, the Constitution laid out the foundations of a welfare state in Brazil. Setting out the blueprints for a welfare state based on universal social rights was no doubt a tremendous achievement, not only on account of other social right systems in the region maintaining their stratified and exclusionary nature, but also on account of the fact that it emerged at a time when the European welfare system was coming increasingly under pressures of various kinds. Having been one of the most organized social movements that led to the overthrow of the military regime and to the new Constitution, the *movimento sanitário* (health care movement) achieved perhaps the most radical institutional rupture in Brazil’s social policy design: universal and equitable health care for all (Art. 196). For the first time, the Brazilian state was called upon to guarantee free and universal health care for nearly 200 million Brazilians through the Unified Health System (*Sistema Único de Saúde*, SUS).

A cursory glance at Brazil’s improving health indicators suggests that the SUS has had a number of notable and important achievements. The most important are in primary/basic health care, prenatal care, vaccination and the free–for–all National AIDS Program, referred worldwide as the “Brazilian AIDS model”. Given the enormity of the SUS, it is not surprising perhaps that it should still suffer from a number of persistent problems, such as gaps in coverage, regional disparities and barriers to accessing specialist and high–complexity care. However, the main challenges to universal health care in Brazil – and indeed elsewhere – are not of an organizational, but primarily of a political economy nature. More specifically, three of the key challenges facing the universal health care system in Brazil are its persistent underfunding, the de–universalization of the right to health and weak pharmaceutical productive capacities needed to sustain it, considered in turn below.

The trajectory of the Brazilian universal health care system has been determined in part by its emergence at a time when the transformation of the Brazilian state along neoliberal lines made its success a particularly challenging task. It is difficult to overlook the fact that whereas health care provisions had been partial and exclusionary during Brazil’s period of fast economic growth, they became constitutionally universal when Brazil’s economic fortunes plunged and its earlier economic successes started to unravel. It is now generally accepted that the period following the debt crisis of the early 1980s up until the 1990s was a “lost decade” for Brazil. Most economic and social indicators deteriorated. For instance, during 1980–1999 GDP grew at around 2.5% per annum – nearly half that of India during this period – and the income of the richest 10% divided by the income of the poorest 10% of the population increased from a factor of 22 in 1960, to 80 in 1989, making Brazil the second most unequal country in the world [3]. The most notable success of the earlier period, the industrial base, became undone as de–industrialization, de–nationalization and deterioration of technological intensity took hold: the share of industry fell as low as 27% of the GDP in the late 1990s, from a high 44% in 1980, and the share of high–tech manufactures represented only 7.9% of Brazil’s total merchandise exports in 2004 compared to 30.5% for China and a 29% world average [4].

Although these developments characterized to a lesser or greater degree most countries in the region, the peculiar way in which they unfolded in Brazil had much to do with the manner in which neoliberal reforms were implemented, especially during the 1990s. What is most relevant to the discussion here is the commitment to neoliberal macroeconomic orthodoxy: the triad of high primary budget surpluses, an inflation–targeting regime and a floating exchange rate with relatively free capital mobility. Despite the shift from neoliberalism to what is often referred to as the neo–developmentalism with the coming in power of the PT (*Partido dos Trabalhadores* – Workers Party) in 2003, this commitment has remained intact. In turn, this continued commitment to neoliberal macroeconomic prescriptions perpetuated a “deadly triad” of overvalued exchange rates, high interest rates and relatively low levels of public and private investment, shifting the level of accumulation toward financialization and commodity extraction/production. In practice, this orientation has resulted in a massive transfer of resources to the financial sector, at the expense of both productive sectors and of social policy. As a result, all three challenges mentioned above – underfunding, the de–universalization of the right to health and weak pharmaceutical productive capacities in health care – have also been perpetuated, raising serious questions about the sustainability of the Brazilian universal health care system in practice.

Underfunding became a problem as soon as the ink on the new Constitution dried. Aiming to bring the social *on par* with the economic, the funding of the universal social security system in Brazil – consisting of social insurance (pensions), health care and social assistance – was to be separate from the fiscal budget. Nonetheless, despite the constitutional principle of integrality of the social security system, in practice, the three areas were separated and, besides, debt repayment took precedence overall. Having already been significantly reduced between 1989–1992, health care funding suffered two additional blows in the early 1990s. First, the main social contribution, payroll taxes, were earmarked for social insurance payments (pensions), thus reducing the funds available for health care. Second, a new Emergency Social Fund was established which, despite its name, allowed the government to direct up to 20% of taxes/contributions toward debt repayment, further reducing funding available for health care. In light of chronic funding shortages in the health care sector, a new tax (CPMF) levied exclusively for this purpose on financial transactions was introduced in 1996, but only about one–third of it was actually used for this purpose, the rest being channeled toward debt repayment and, later, to other social assistance programs [5].

Although GDP grew at an average annual rate of 4.5% during the 2004–2010 (neo–developmentalist) period, the issue of underfunding of the health care sector was not resolved. Federal social spending increased from 12.6% to 15.8% of the GDP between 2000 and 2009, but nearly half of it was claimed by social insurance (pensions), as had happened during the 1990s [6]. Likewise, the 30% share of Social Security Budget committed to health care was never respected and the ‘de–earmarking’ mechanism regularly channeling funds from it to debt repayment continued. Besides, the 1996 financial transaction tax collected specifically to fund health care, although never exclusively used for this purpose, was completely dissolved in 2007 and no new taxes or financial instruments have been put in place to address the chronic shortfall in the sector. The result has been that *federal* spending on health care remained practically unchanged from 1995 onwards at around 1.8% of the GDP, and the total public health care expenditure rose only from 3.2% in 2003 to 3.9% of GDP in 2012 [7]. This is higher than the 1.14% equivalent share in India in 2012, but still constitutes less than half the 8.3% average in countries with a similar commitment to universal health care.

That the financial base of the Brazilian health care system is incompatible with the constitutional commitment to universality is also visible in the low share of *public* health care expenditure which continued to be below 50% of the total health care expenditure during 1990s and 2000s, compared to a minimum of 70% for other universal health systems. This has resulted in a situation where less than 30% of Brazilians who continue to use private health insurance and facilities constitute more than 50% of the total health care expenditure in Brazil. In India, the private sector today provides nearly 80% of outpatient care and about 60% of inpatient care [8], but presumably many in need of health care do not appear as patients of any kind in government’s statistics. What the Brazilian figures suggest is that the legacies of the previous discriminatory health care system are still in operation and the constitutional principle of universality is yet to be realized in practice. This is in fact a wider problem that relates to the de–universalization of the social rights in general. Indeed, although social spending grew during the 1990s, it remained woefully inadequate to support the universal social security rights guaranteed by the Constitution. Social policy during this period was one of ‘inclusive liberalism’ whereby various conditional cash transfer programs targeting the poorest sabotaged the achievement of universal social rights guaranteed by the Constitution, including that of health. It is true that the neo–developmental state achieved some remarkable successes in social policy, the most important of which have been the reduction of wage inequality, the rise of the (real) minimum wage and the rise of income of the poorest, especially via targeted social programs such as *Bolsa Família.* Although poverty levels fell and the Gini coefficient was reduced for the first time in decades, income inequality remained high: in 2007, the income shares of the poorest and richest 10% were 0.9% and 44%, respectively [9]. More importantly, the tendency of social spending to reinforce in some respects the de–universalization of social rights that was put in train during the previous decade was not reversed. On the contrary, the strong expansion of *private* social services and the continued preference for conditional cash transfers targeting the poorest continued to compromise the constitutional universality of social rights.

The challenges of underfunding and de–universalization of the right to health in practice stem in large part from the contradictions between a neoliberal monetary policy and a neo–developmentalist social policy. One way in which these contradictions manifest themselves can be grasped by the following figures: around 8.1% of the GDP was handed out to domestic and foreign creditors as debt repayments in 2005, compared to a modest 3.3% of the GDP on public health care expenditure, and a dismal 0.3% of GDP toward the flagship *Bolsa Família* program [7]. It must be added that contradictions exist not only between macroeconomic and social policies, but industrial policies, too. No substantial industrial policy measures were taken during the 1990s in Brazil. As mentioned earlier, this period was characterized by de–industrialization, de–nationalization and falling technological intensity. This broader context had negative consequences for the national health–pharmaceutical sector. Instead of thriving at the time the SUS was being rolled out, the domestic health–pharmaceutical system that had been weak and dominated by imports and foreign pharmaceutical companies earlier on, became even weaker during this period so that only one among the top 20 pharmaceutical companies in Brazil in the mid–1990s was nationally–owned [10]. One outcome of such weak national productive capacities was the rapidly increasing trade deficit of the health care sector, growing from US$2.4 billion in 2003 to just over US$ 10 billion in 2012, half of which was accounted for by the deficit in the pharmaceutical sector alone [7].

The fortunes of the national pharmaceutical sector took a favorable turn with the introduction of the generics category in the market following the creation of a new agency in 1999 (ANVISA) and, after the rise of the neo–developmental state in 2003, with the health–pharmaceutical sector singled out as one of four strategic sectors in its first industrial policy. Following the focus of industrial policies on the sector, considerable funds have been channeled toward the health–pharmaceutical sector, primarily via the BNDES Profarma Program, largely on enhancing productive capacities, but innovation, too. As a result of these and other measures, the share of generics markets in Brazil grew to 17% of the total market in 2008, of which 88% was controlled by Brazilian firms [10]. Although it is too early to evaluate the outcome of these recent efforts, it must be noted that the lack of rules aimed at controlling foreign ownership has contributed to a new wave of acquisitions has already seen some of the emerging/successful domestic companies bought by foreign pharmaceutical companies, a development that does not bode well for the success of Brazil’s universal health care system. 48 transnational pharmaceutical companies still account for around 80% of the total market (by revenues), followed at a long distance by public laboratories and private Brazilian companies, contributing to a growing sectoral trade deficit.

**Figure:**
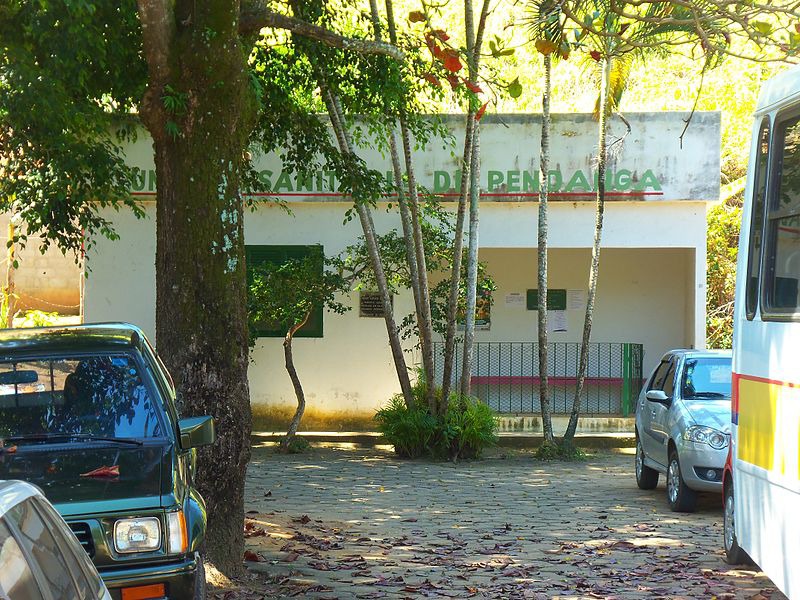
Photo: Unidade Sanitária Rural de Pendanga, Ibiraçu (Brazil) – a rural health clinic in Brazil. Via Wikimedia Commons Creative Commons Attribution–Share Alike 3.0 Unported.

The challenges facing Indian policymakers in their efforts to build a national health care system will no doubt be many and finding ways to meet them can only partially be helped by observing the trajectory of similar efforts elsewhere. At the very least, this brief discussion indicates that unless social, macroeconomic and industrial policies are co–articulated and directed toward serving the needs of the society as a whole, a universal health care system in a country with high income concentration like India and Brazil risks becoming an inferior subsystem that attends predominantly but inadequately to the poorer segments of society. Political will and persistence is crucial; in the case of Brazil, for instance, the recent removal of the PT–led government and its replacement by a neoliberal–minded one may weaken the fragile foundations of Brazil’s universal health care system. The key challenges to building and maintaining a successful universal health care system have always been and remain of a political economy nature.
